# Analyzing canopy structure effects based on LiDAR for GPP-SIF relationship and GPP estimation

**DOI:** 10.3389/fpls.2025.1561826

**Published:** 2025-05-19

**Authors:** Shuo Shi, Zixi Shi, Fangfang Qu, Wei Gong, Lu Xu, Chenxi Liu

**Affiliations:** ^1^ State Key Laboratory of Information Engineering in Surveying, Mapping and Remote Sensing, Wuhan University, Wuhan, Hubei, China; ^2^ Collaborative Innovation Center of Geospatial Technology, Wuhan Hubei, China; ^3^ Perception and Effectiveness Assessment for Carbon-neutrality Efforts, Engineering Research Center of Ministry of Education, Wuhan, Hubei, China; ^4^ Wuhan Institute of Quantum Technology, Wuhan, Hubei, China; ^5^ Electronic Information School, Wuhan University, Wuhan, China; ^6^ School of Geology and Geomatics, Tianjin Chengjian University, Tianjin, China

**Keywords:** GPP, LiDAR, SIF, canopy structure, GEDI

## Abstract

The coupling between Gross Primary Productivity (GPP) and Solar-Induced Chlorophyll Fluorescence (SIF) is crucial for understanding terrestrial carbon cycles, with the GPP/SIF ratio regulated by canopy structure, environmental change, and other factors. While studies on canopy structure focus on how internal structure regulates light use efficiency, the impact of remotely sensed canopy structural parameters, particularly Fractional Vegetation Cover (FVC) and Leaf Area Index (LAI), on GPP-SIF coupling remains understudied. Investigating the response of canopy structure to GPP-SIF in large-scale forests supports high-accuracy GPP estimation. LiDAR offers unparalleled advantages in capturing complex vertical canopy structures. In this study, we used multi-source data, particularly LiDAR-derived canopy structure products, to analyze the annual variations in canopy structural parameters and GPP/SIF across different forest types, investigate the response of canopy structure to the GPP-SIF relationship, and employ machine learning models to estimate GPP and assess the contribution of canopy structural factors. We found that LiDAR-derived canopy structure products effectively captured vegetation growth dynamics, exhibiting strong correlation with MODIS products (maximum R²=0.95), but with higher values in densely vegetated areas. GPP/SIF exhibited significant seasonal and forest-type variations, peaking in summer. Its correlation with canopy structural parameters varied seasonally, ranging from 0.21 to 0.75. In summer, the correlation decreased by 5.53% to 30.59% compared to other seasons. In random forest models, incorporating canopy structural parameters improved GPP estimation accuracy (R^2^ increasing by 1.30% to 8.07%).

## 1 Introduction

Terrestrial gross primary production (GPP), representing the amount of atmospheric carbon dioxide (CO_2_) fixed by vegetation photosynthesis, constitutes the largest flux of the global carbon budget and plays a fundamental role in the carbon cycle (Liao et al., 2023; Ryu et al., 2019; Wang et al., 2021a). Accurately quantifying GPP is of great significance for monitoring forest growth conditions (Xie et al., 2019). The utilization of remote sensing techniques for large-scale, high-precision GPP estimation and subsequent forest state assessment has become a research hotspot.

Solar-induced chlorophyll fluorescence (SIF) is a radiation flux driven by APAR, emitted as a light in the spectral range of 650–800nm (Mohammed et al., 2019). Studies have confirmed that SIF is strongly correlated with GPP, making it a direct and reliable indicator of GPP (Cheng et al., 2021; Pierrat et al., 2022; Wu et al., 2022a; Yang et al., 2018). Compared to traditional vegetation indices, SIF exhibits a stronger ability to monitor terrestrial photosynthesis (Li et al., 2018). Satellite-based SIF observations have been widely applied as a proxy for GPP at the global scale, further demonstrating its effectiveness in large-scale carbon cycle studies (Wang et al., 2021a).

While SIF has been widely used as a proxy for GPP, the GPP-SIF relationship is not uniform across different conditions, leading to uncertainties in GPP estimation. This relationship varies significantly across different vegetation types and growing seasons (Bai et al., 2022; Chen et al., 2021b). Studies have shown that the GPP-SIF relationship is influenced by biomes, climate conditions, and canopy structure (Guanter et al., 2012; Sun et al., 2017; Wang et al., 2021b). Especially, canopy structure plays a crucial role in the GPP-SIF relationship. Zhang et al. (2019) found that the canopy-leaving SIF observed by sensors represented only a portion of the total canopy SIF emission, which was sensitive to canopy structure and observation direction. Correcting for canopy structure helped improve the consistency between SIF and GPP. Dechant et al. (2020) analyzed the GPP-SIF relationship from a mechanistic perspective, demonstrating the dominant role of canopy structure and establishing a strong link between the near-infrared reflectance of vegetation (NIRV) and the canopy structure information embedded in the SIF signal. Yazbeck et al. (2021) demonstrated that canopy structure played a dominant role in the GPP-SIF relationship across timescales, with a stronger influence at longer timescales. Incorporating canopy structural variables into the SIF-GPP relationship improved site-level GPP predictions and reduced estimation uncertainty. Existing studies have mechanistically emphasized the influence of canopy structure on the GPP-SIF relationship at the site scale. However, it remains unclear whether this relationship holds at larger scales, especially in complex forest canopies. Additionally, the influence of product-level canopy structure data on large-scale GPP-SIF estimations from remote sensing is still underexplored.

In commonly acquired canopy structure products from remote sensing technology, fractional vegetation cover (FVC) and LAI are critical in canopy radiative transfer and photosynthetic process (Fang et al., 2021; Gao et al., 2020). As important indicators of canopy, they have been widely applied in researches. LAI has been shown to have a strong response to SIF and GPP (Bai et al., 2022; Wu et al., 2022b; Xie et al., 2019; Zhang et al., 2021). FVC has a large influence the SIF emission (Zeng et al., 2020). Escaping probability of canopy SIF and total emitted SIF showed nonlinear responses to FVC (Zhang et al., 2022). Despite their importance, the roles of FVC and LAI in the GPP-SIF relationship remain underexplored.

At large scales, the accuracy of canopy structure products also affects the reliability of conclusions regarding GPP-SIF responses. Currently, large-scale canopy structure products are primarily derived from passive remote sensing data (Fang et al., 2019). Using LAI as example, various global LAI products based on optical imagery have been found obvious inconsistencies and uncertainties due to the vegetation characterization, input data and retrieval algorithms (Breunig et al., 2011; Camacho et al., 2013; Fang et al., 2013; Jiang et al., 2017; Jin et al., 2017; Xiao et al., 2017). Due to limitations in measurement principles, light saturation effects of radiation at high LAI can lead to underestimation of LAI (Liu et al., 2012). Especially in forested areas, the underestimation of canopy structure is significant due to the inability of passive optical sensors to penetrate beneath the canopy (Li et al., 2022; Xu et al., 2024). Moreover, current global LAI retrieval algorithms largely neglect topographic effects, introducing uncertainty into GPP research and resulting in better simulation performance in flat than in complex terrain (Xie et al., 2019). LiDAR, as an active sensor, has the ability to penetrate forest canopies, offering a distinct advantage in quantitatively assessing forest ecosystems (Moran et al., 2018; Shi et al., 2025). In 2018, NASA launched the GEDI (Global Ecosystem Dynamics Investigation LiDAR) mission, equipped with a full-waveform LiDAR (Dubayah et al., 2020a). GEDI has been used to monitor forest growth conditions, generating public multi-level canopy structure products, which offered a new source for GPP inversion research and hold significant potential for widespread applications (Tang et al., 2019). LiDAR help reduce estimation errors caused by topography and light saturation, enabling accurate retrieval of canopy structral parameters in densely vegetated areas, facilitating a more precise assessment of its impact on GPP estimation and the GPP-SIF relationship. Currently, there is a lack of research in the field of GPP-SIF relationship using LiDAR data.

In this study, we integrated spaceborne LiDAR data and multi-source data across five forest types in the continental United States to investigate how canopy structral parameters (LAI and FVC) influence the GPP-SIF relationship and GPP estimation in large-scale. We addressed the following questions: (1) How do the seasonal variations of GPP/SIF and LiDAR-derived canopy structure differ across tree species? (2) How do LiDAR-based canopy structure products respond to GPP/SIF? (3) What are the contributions of LAI and FVC to large-scale GPP estimation using machine learning models?

## 2 Materials and methods

### 2.1 Data collection

**Figure 1:**
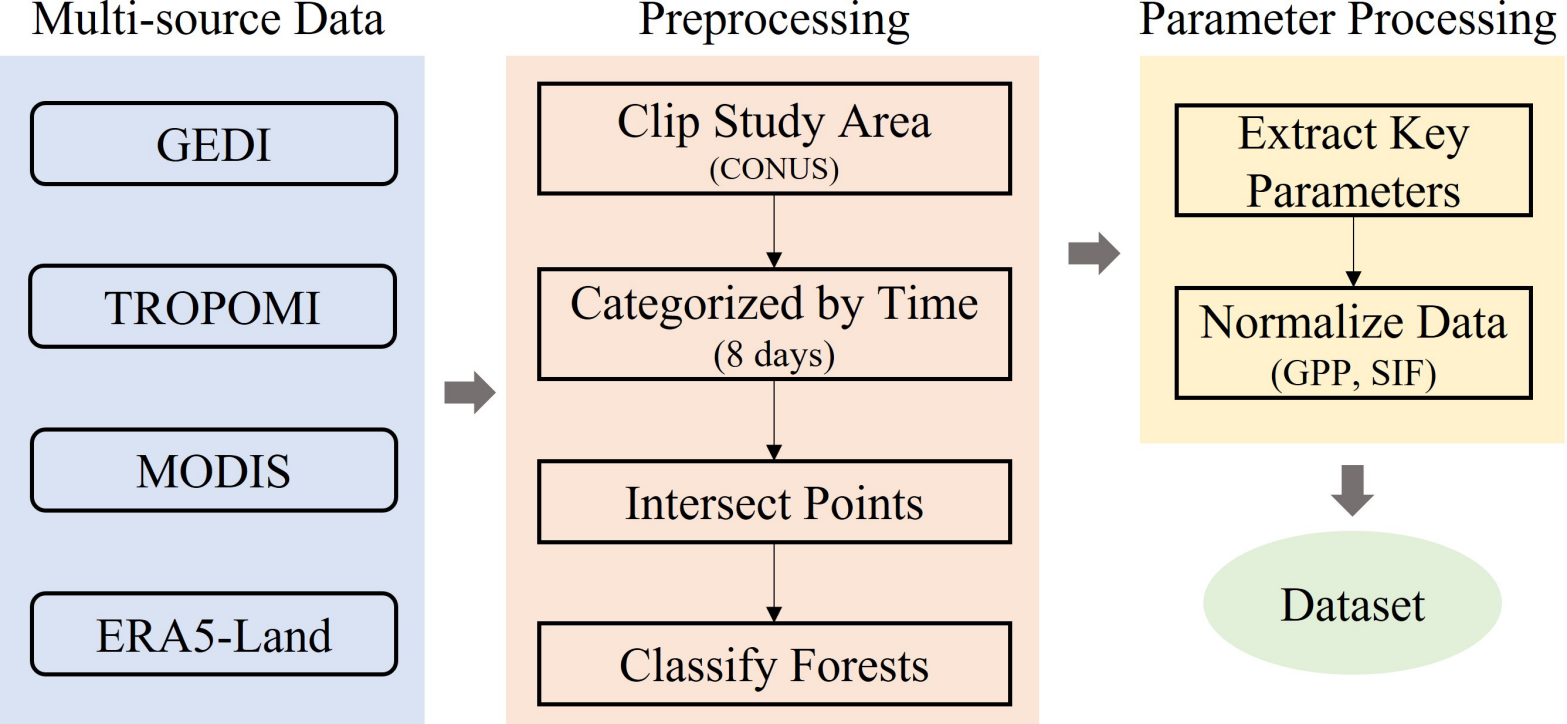
Multi-source data and data preprocessing flow of this study.

Various remote sensing data with an 8-day resolution were collected to obtain parameters related to canopy structure and productivity across the continental United States (CONUS). Especially, in calculating GPP/SIF, we normalized GPP and SIF products to fall within the [0,1] range. The normalization method has been proved not to affect the result (Chen et al., 2021b). **Figure 1** showed the multi-source remote sensing data and data preprocessing flow of this study.

#### 2.1.1 Canopy structure metrics

The Global Ecosystem Dynamics Investigation (GEDI), as a new spaceborne LiDAR instrument launched in December 2018, was specifically designed to measure high resolution 3D vegetation structural metrics and estimate biomass under different environmental conditions (Dubayah et al., 2020a; Liu et al., 2021a). The GEDI instrument is comprised of 3 lasers illuminating a 25 m spot (a footprint) on the surface and each footprint is separated by 60 m along track, with an across-track distance of about 600 m between each of the 8 tracks (Dubayah et al., 2020a; Potapov et al., 2021). The GEDI Level 2 data products include L2B Canopy Cover and Vertical Profile Metrics, available from the NASA’s Land Processes Distributed Active Archive Center (LPDAAC) (Dubayah et al., 2020b). In this study, we acquired the GEDI L2B (version 1.0) products and extracted FVC and LVC data.

For data assessment and comparison, we also obtained LAI and FVC data from MODIS products. LAI was acquired from MCD15A3H Version 6 LAI product (4-day composite data set with 500-meter pixel size). FVC was derived from MOD13A1 Version 6 product Enhanced Vegetation Index (EVI) values (16-day composite data set with 500-meter pixel size). The calculation Equation 1 is as follows:


(1) $$\begin{array}{c} FVC=\frac{EVI-EVI_{s}}{EVI_{v}-EVI_{s}} \end{array}$$

$EVI_{s}\;$ is the mean 5% value of total pixels, and $EVI_{v}$ is the mean 95% value of total pixels.

In this experiment, we aimed to standardize the temporal resolution of the data to an 8-day interval. Due to the 16-day temporal resolution of MODIS vegetation index products, some MODIS FVC data were partially missing as a result.

#### 2.1.2 SIF data

The Tropospheric Monitoring Instrument (TROPOMI), launched as the single payload of ESA’s Sentinel-5 Precursor (S-5P) satellite in 2017, is enabled to monitor terrestrial SIF (TROPOSIF) (Borsdorff et al., 2018). TROPOMI combines a global continuous spatial sampling with a 3.5×7.5 km^2^ pixel size at nadir in the near infrared (3.5×5.5 km^2^ since August 2019) with a daily revisit time (Butz et al., 2012). The L2B TROPOSIF product consists in global daily SIF_743_ and SIF_735_ in netCDF-4 files (Guanter et al., 2015). In this study, we acquired the L2B TROPOSIF product covering the CONUS for the whole year of 2020 and the SIF data was summarized into one set for every eight days.

#### 2.1.3 GPP data

We selected two types of GPP data: flux tower data and remote sensing products, to conduct a more accurate assessment of results. We obtained weekly AmeriFlux FLUXNET (CC-BY-4.0) SUBSET data from 33 forest flux towers across the CONUS, covering the year 2020. The obtained GPP values (GPP_DT_VUT_REF) from these sites were denoted as “GPP_AMF_”. The 33 flux towers represented the following forest types: 15 deciduous broadleaf forests (DBF), 15 evergreen needleleaf forests (ENF), and 3 mixed forests (MF). The flux tower for the other two forest types deciduous needleleaf forests (DNF) and evergreen broadleaf forests (EBF) were not found.

The GPP product used in this paper is MOD17A2H Version 6, a cumulative 8-day composite of GPP values with 500-meter (m) pixel size based on the radiation-use efficiency concept. The MODIS Adaptive Processing System (MODAPS) at the NASA Goddard Space Flight Center produces this gridded Collection-5 GPP product, which is validated to Stage-3 (Running et al., 2015). In this experiment, we obtained MOD17A2H GPP data every 8 days in 2020 covering the CONUS (denoted as “GPP_MOD_”).

#### 2.1.4 Land cover data

Accurate extraction of vegetation land cover is essential for forest research. The MCD12C1 Version 6 data product provides maps of the International Geosphere-Biosphere Programme (IGBP) classification schemes at yearly intervals at 0.05 degree (5,600 meter) spatial resolution for the entire globe from 2001 to 2020 (Friedl and Sulla-Menashe, 2015). Here, we selected 5 major forest types based on the IGBP classification scheme including evergreen needleleaf forest (ENF), evergreen broadleaf forest (EBF), deciduous needleleaf forest (DNF), deciduous broadleaf forest (DBF) and mixed forest (MF).

#### 2.1.5 Environmental parameters

Environmental factors such as temperature, humidity, and radiance have been shown to affect vegetation productivity (Gonsamo et al., 2016). We got the environment parameters from a reanalysis dataset ERA5-Land (Muñoz Sabater, 2019). It has been produced by replaying the fifth-generation global land component of the European Centre for Medium Range Weather Forecasts (ECMWF) ERA5 climate reanalysis with a 0.25° spatial resolution. In this study, we downloaded ERA5-Land daily aggregated meteorological data including the 2 m temperature $T_{a}$ ( °C ), 2 m dewpoint temperature $T_{d}$ ( °C ) and daily surface downward solar radiation SW ( $\text{W}/{\text{m}}^{2}$ ) in 2020. Then, we used $T_{a}$ and $T_{d}$ to calculate vapor pressure deficit VPD ( hPa ) (Bai et al., 2022; Zheng et al., 2020). In this experiment, the obtained $T_{a}$ , VPD , and SW data would be used as input parameters for the GPP inversion model.

### 2.2 Methods

**Figure 2:**
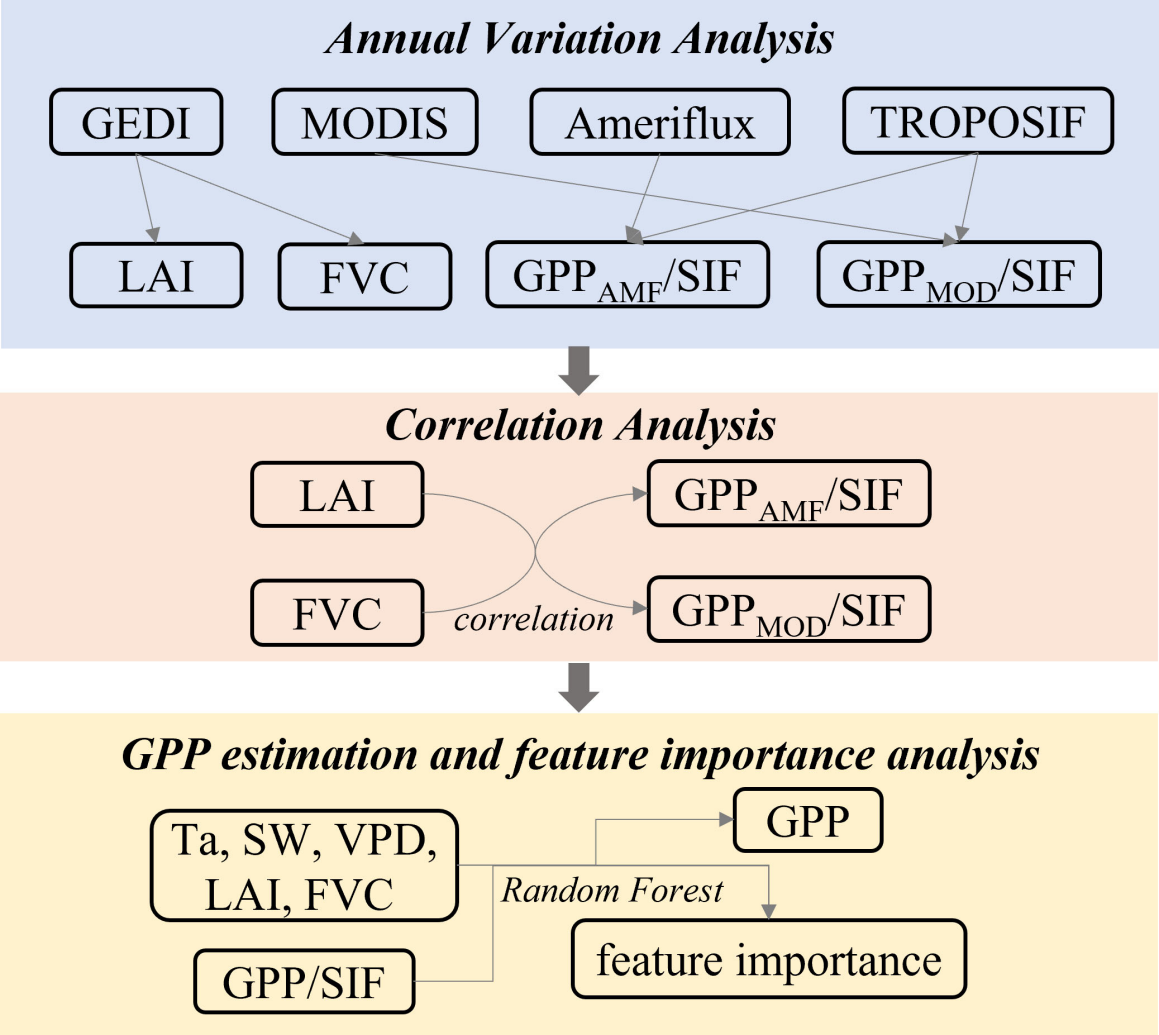
The research methods and procedures employed in this study.

**Figure 2** illustrated the research methods and procedures employed in this study. Firstly, we used mathematical and statistical methods to analyze annual variations in GEDI and MODIS canopy structure products, as well as GPP/SIF values (including GPP_AMF_/SIF and GPP_MOD_/SIF) across different forests. We assessed the quality of GEDI data by comparing it with MODIS products, identifying changes in canopy structure and GPP/SIF for each forest type.

Secondly, in order to investigate the relationship between canopy structure and GPP/SIF (including GPP_AMF_/SIF and GPP_MOD_/SIF), quantitative approaches were adopted. We conducted correlation analysis between canopy structural parameters and GPP/SIF every 8 days. Pearson correlation coefficient (Cohen et al., 2009) was calculated for different forest types.

Finally, we assessed the contribution of structural parameters to the calculation of GPP_MOD_/SIF and the subsequent inversion of GPP using random forest. We incorporated various features as inputs to the random forest (RF) model (Rigatti, 2017) to estimate GPP_MOD_/SIF. Then the GPP was estimated using the SIF and estimated GPP_MOD_/SIF. Based on random forest model RF1 and RF2, we estimated GPP using different sets of input feature parameters. RF1 included canopy structural parameters, while RF2 did not. The GPP inversion Equations 2–3 are as follows:


(2) $$\begin{array}{c} GPP_{RF1}=f\left(LAI,FVC,T_{a},SW,VPD\right)\times SIF \end{array}$$

(3) $$\begin{array}{c} GPP_{RF2}=f\left(T_{a},SW,VPD\right)\times SIF \end{array}$$

where LAI is leaf area index,  FVC is the fractional vegetation cover, $T_{a}$ is the air temperature ( °C ), SW is the surface downward solar radiation ( $\text{W}/{\text{m}}^{2}$ ), VPD is the vapor pressure deficit ( hPa ).

The model was implemented in Python 3.12 using the ‘RandomForestRegressor’ function from the scikit-learn 0.24.1 package to develop two random forest models, RF1 and RF2. Except for differences in input data, both models were configured with identical parameters, specifically: the number of decision trees (n_estimators) was set to 150, Out-of-Bag (OOB) evaluation was enabled, and the random seed (random_state) was fixed at 22 for reproducibility, while all other parameters remained at their default settings. The computational environment consisted of an Intel Core i5-13600K CPU (3.50 GHz) and an NVIDIA T400 4GB GPU.

The metrics used to evaluate the accuracy of GPP inversion included the goodness of fitting and generalization ability of the random forest model, as well as the coefficient of determination (R^2^) and root mean square error (RMSE) between the predicted values and the true values. The principles of each evaluation metric refer to the python ‘RandomForestRegressor’ documentation.

Feature importance analysis for the trained random forest model was conducted using the ‘permutation_importance’ from the scikit-learn 0.24.1 package. The ‘n_repeats’ was set to 5, permuting each feature five times to average its impact on model performance. ‘random_state’ was set to 0 for reproducibility, with all other parameters at default settings.

## 3 Results

### 3.1 Annual variation of LiDAR canopy structural parameters

**Figure 3:**
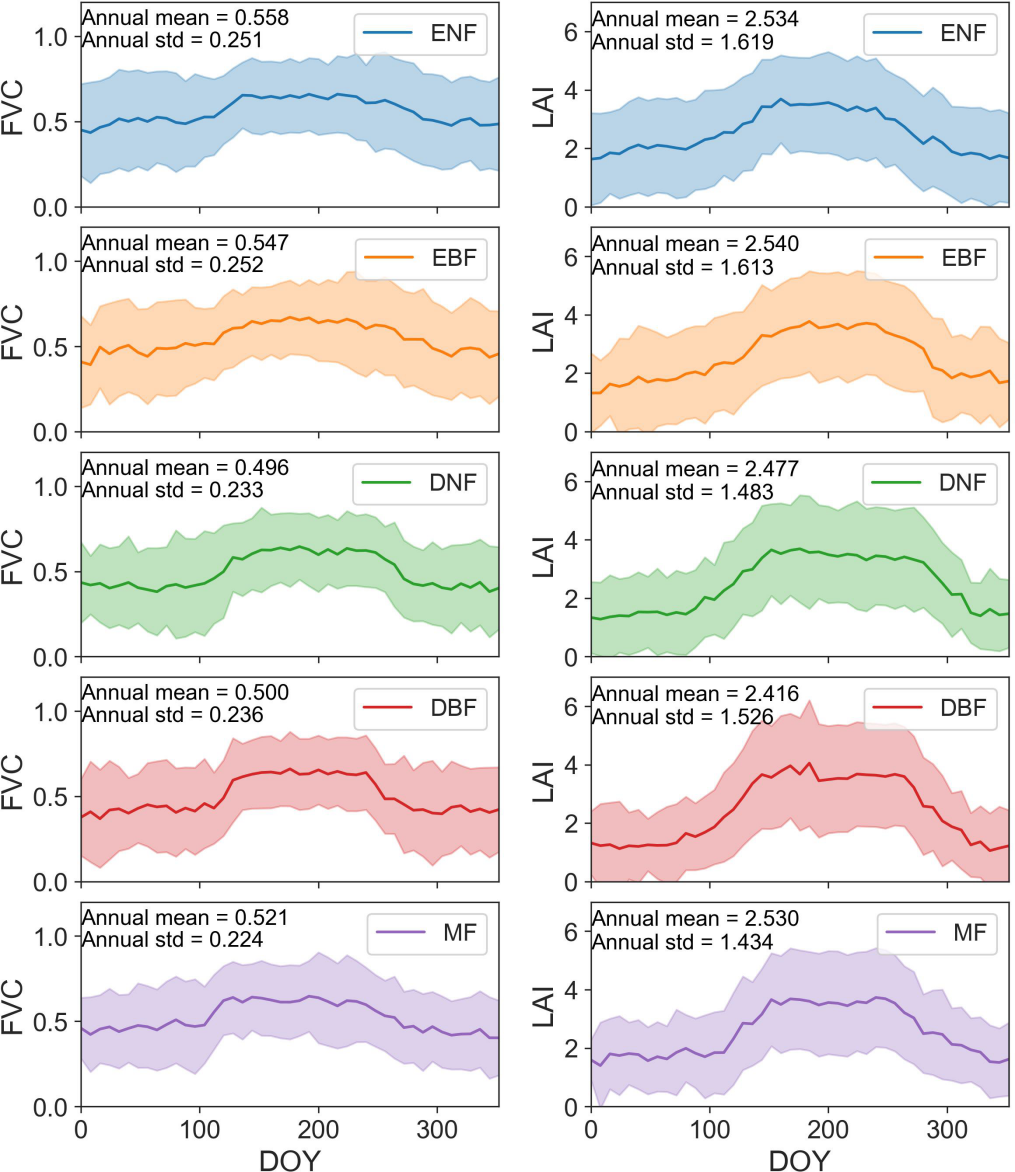
The average FVC and LAI of five forest types in CONUS (the error bars represent the standard deviation of each set of data).

The average of FVC and LAI obtained from GEDI for five types of forests were calculated (**Figure 3**). The results revealed significant differences in the FVC values among different forests. All forests exhibited a general pattern of increasing followed by decreasing average FVC trends throughout the year. Among them, the magnitude of FVC variation followed the order: DBF>DNF>MF>EBF>ENF, indicating a higher degree of variation in deciduous forests compared to evergreen forests and a higher variation in broadleaf forests compared to needleleaf forests. In the non-summer seasons, the total average FVC values ranked as follows: ENF>EBF>MF>DNF>DBF. During the summer season, the order of total average FVC values was: EBF>ENF>DBF>MF>DNF. For LAI, the values exhibited a similar trend to FVC, with higher values during the summer compared to other seasons. Comparing the annual variation in average LAI, the magnitude of change was ranked as follows: DBF>DNF>MF>EBF>ENF, indicating that deciduous forests showed a greater overall variation than evergreen forests. In non-summer seasons, the ranking of average LAI values was ENF>EBF>MF>DNF>DBF. During the summer, the average LAI followed the order: DBF > DNF > EBF > MF > ENF, with DBF having the highest average 3.515 and ENF having the lowest value of average 3.322.

**Figure 4:**
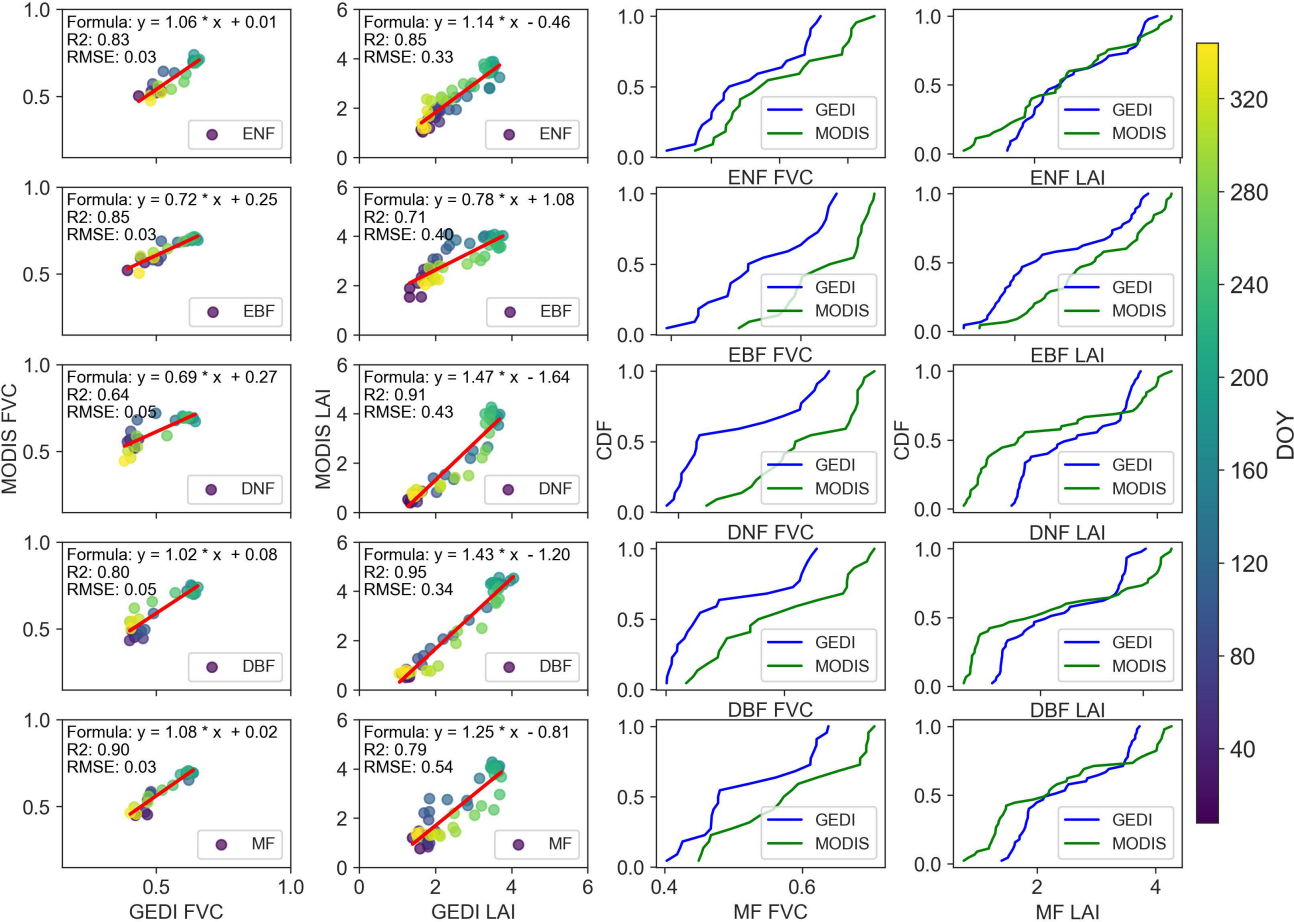
Comparison of GEDI and MODIS derived FVC and LAI across different forest types: regression analysis and cumulative distribution function (CDF) evaluation.

To further assess GEDI data, we obtained MODIS products at an 8-day interval and conducted a correlation analysis on the annual variations of their means (**Figure 4**). The results showed high consistency between MODIS and GEDI in annual FVC and LAI variations across five forest types. The correlation R^2^ for FVC ranged from 0.64 (DNF) to 0.90 (MF) and for LAI from 0.71 (EBF) to 0.95 (DBF). Seasonal analysis revealed summer data concentrated centrally, with other seasons at the extremes, indicating a common trend of increasing then decreasing canopy structural parameters in both datasets. The cumulative distribution function (CDF) results in the third and fourth columns indicated significant differences between the GEDI and MODIS datasets. In most cases, GEDI-derived values (blue) were higher than MODIS-derived values (green) in the lower quantiles, while also exhibiting distinct distribution shapes and spreads. GEDI-derived FVC values were generally shifted toward higher values compared to MODIS, whereas the LAI distributions showed more pronounced differences. Specifically, GEDI exhibited a broader spread, suggesting greater variance in estimated LAI values relative to MODIS.

### 3.2 GPP/SIF changes of different forest types

**Figure 5:**
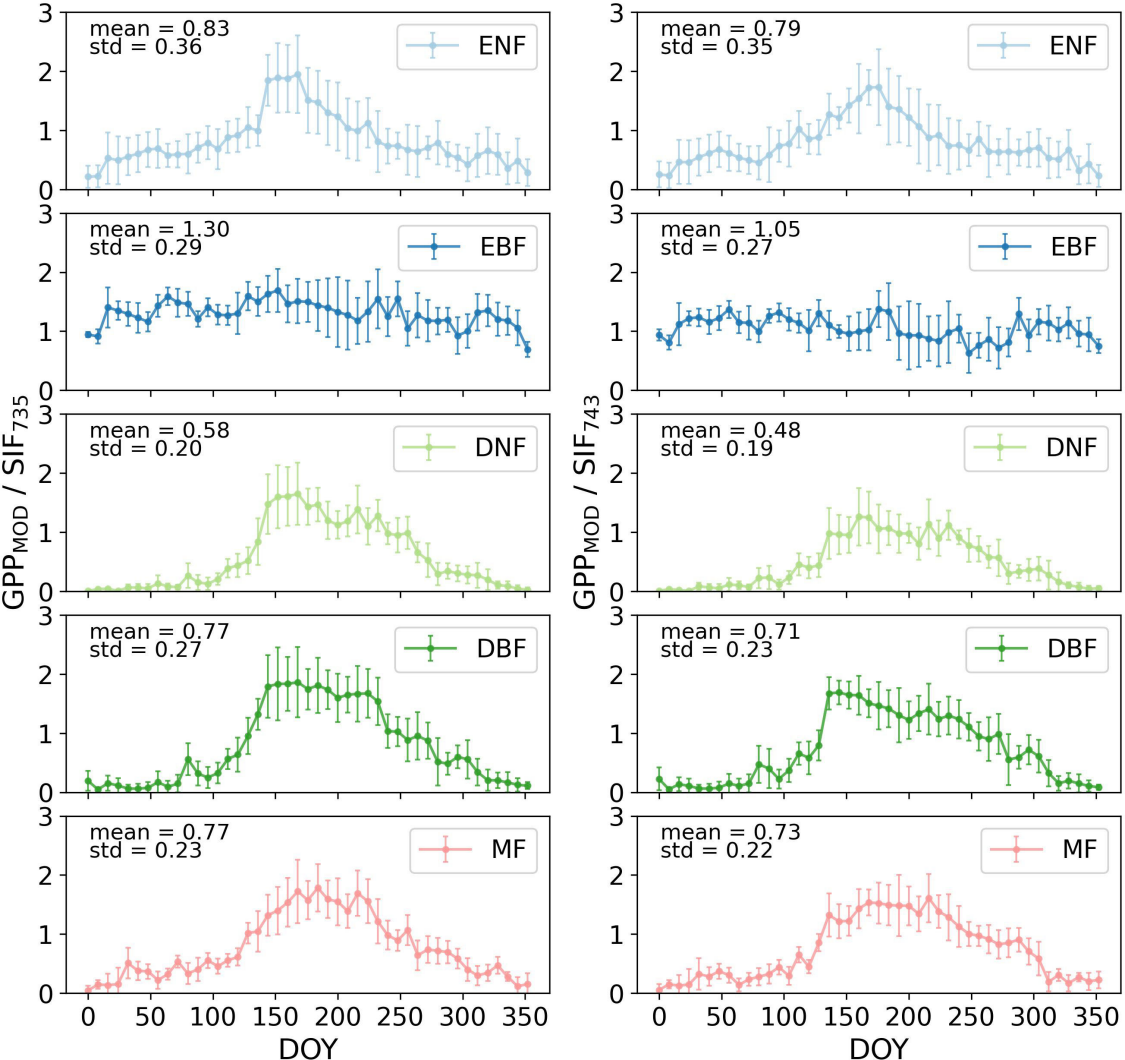
The GPP_MOD_/SIF of five forest types throughout the year 2020 in CONUS (the error bars represent the standard deviation of each set of data).

The GPP_MOD_/SIF of five forest types was shown in **Figure 5**. Among the forests, only EBF did not show a clear pattern of variation in GPP_MOD_/SIF values throughout the year. ENF, DNF, DBF and MF exhibited varying degrees of an increasing followed by a decreasing trend in their ratios, with the ratios during the summer months (June, July, and August) significantly higher than in other seasons.

A detailed analysis revealed that GPP_MOD_/SIF for ENF initially increased and then decreased throughout the year, peaking at 1.95 in June and ranging between 0.5 and 1.0 in the off-summer months. For EBF, the annual mean GPP_MOD_/SIF was 1.30, fluctuating between 1.0 and 1.5 with no significant seasonal variation. DNF showed a clear increasing-then-decreasing pattern, with GPP_MOD_/SIF values nearly negligible from January to April, peaking in May, remaining above 1.0 in summer, and decreasing to near-zero in autumn and winter. DBF exhibited a similar trend, with the highest mean during summer and the most significant variation. Deciduous forests (DNF, DBF) had greater GPP_MOD_/SIF variation compared to evergreen forests (ENF). MF also followed an increasing-then-decreasing pattern, similar to broadleaf forests, but with slightly lower variation.

**Figure 6:**
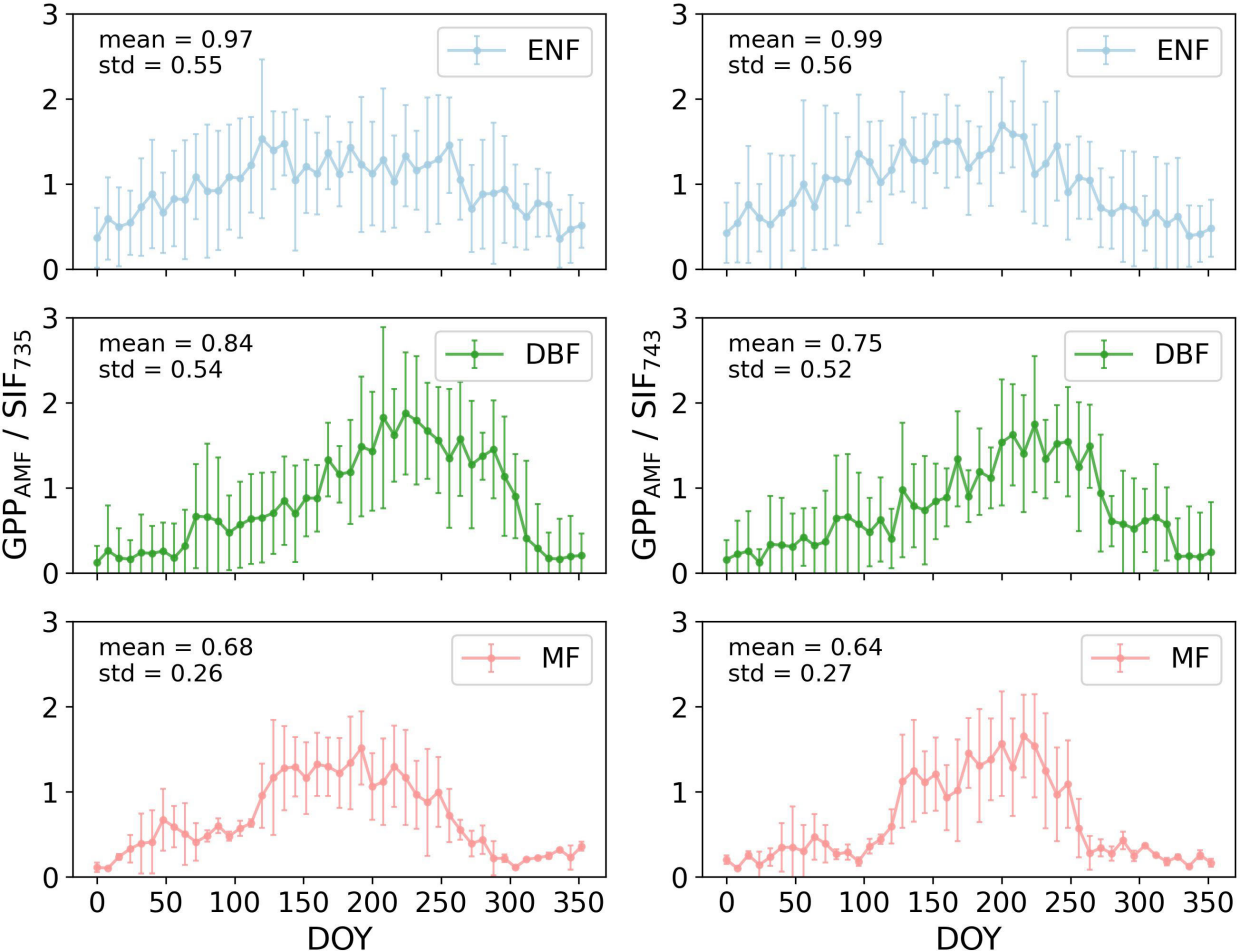
The GPP_AMF_/SIF of three forest types throughout the year 2020 in CONUS (the error bars represent the standard deviation of each set of data).

**Figure 6** illustrated the annual variations of GPP_AMF_/SIF for three forest types based on flux tower data, showing similar increasing-decreasing trends as **Figure 5**, with peaks notably higher in summer. ENF exhibited a smaller annual variation range compared to DBF and MF. The mean GPP_AMF_/SIF values for ENF and DBF were higher than GPP_MOD_/SIF, while MF had a lower mean. Additionally, the standard deviation of GPP_AMF_/SIF was greater than that of GPP_MOD_/SIF, indicating higher annual variation.

### 3.3 Annual correlation between canopy structure and GPP/SIF

**Figure 7:**
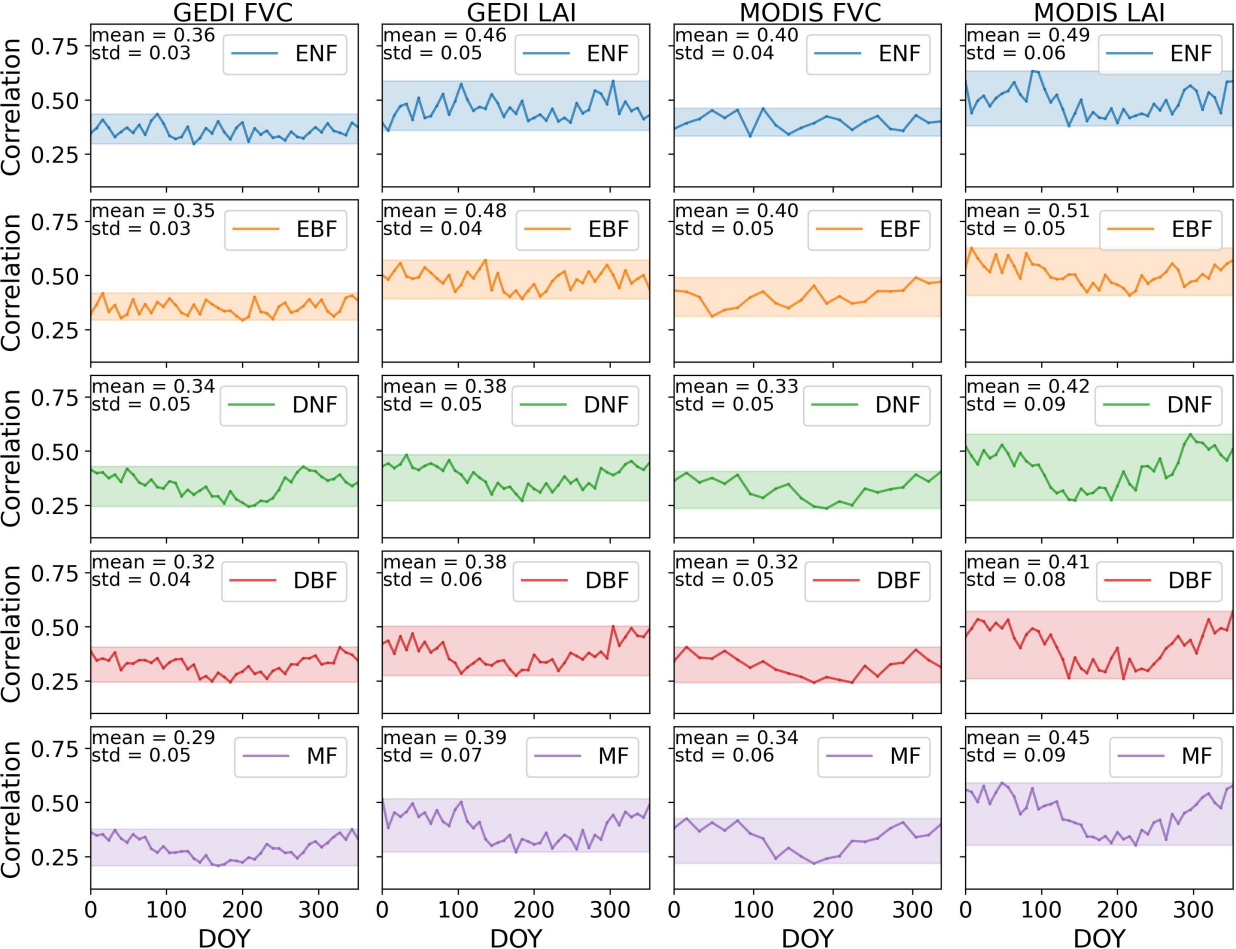
The Pearson correlation coefficients between GPP_MOD_/SIF and LAI, FVC for five forest types across the CONUS in 2020.

**Figure 7** depicted the Pearson correlation coefficient between GPP_MOD_/SIF and LAI, FVC, respectively, for five forest types across the CONUS in 2020. Linear correlation was observed between canopy structural parameters and GPP_MOD_/SIF, with correlations ranging from 0.21 to 0.49 for FVC and from 0.26 to 0.63 for LAI. Compared to MODIS data, GEDI data exhibited relatively lower correlations with GPP_MOD_/SIF, with a more stable changes of annual value.

Distinct patterns emerged in terms of the total correlation and its temporal dynamics across different forests. The mean annual correlation from high to low was: EBF>ENF>DNF>MF>DBF. Evergreen forests showed higher correlations compared to deciduous forests, particularly in summer. The canopy structural parameters of evergreen forests maintained relatively stable values throughout the year. However, deciduous and mixed forests exhibited a noticeable trend of decreasing correlation throughout the year, followed by an increase. In summer, the average correlation of FVC was lower than other seasons, with the decrease magnitude 16.43%-28.44%. The average correlation of LAI was 18.31% to 30.59% lower compared to other seasons.

**Figure 8:**
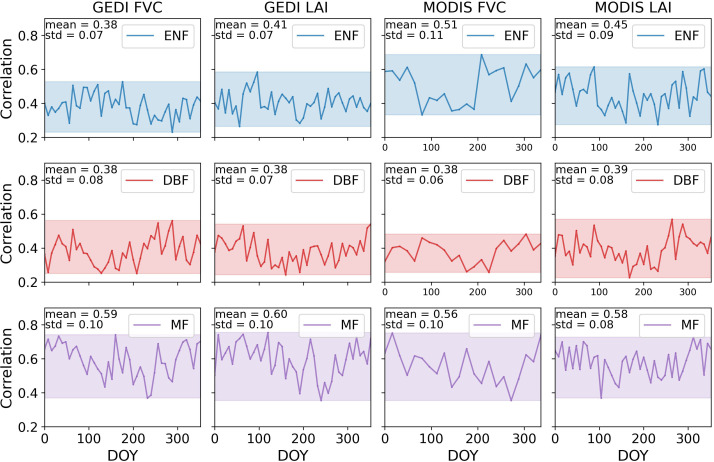
The Pearson correlation coefficients between GPP_AMF_/SIF and LAI, FVC for three forest types across the CONUS in 2020.

**Figure 8** showed the correlation results between GPP_AMF_/SIF and MODIS and GEDI canopy structural parameters, with correlations ranging from 0.22 to 0.75. No significant difference was found between MODIS and GEDI data. Except for GEDI FVC in ENF, the average correlation between FVC and GPP_AMF_/SIF was 6.17% to 13.44% lower in summer compared to other seasons. For LAI, the decrease ranged from 5.53% to 15.18%. These results highlight the variation in LAI, FVC, and GPP/SIF correlations among different forest types, particularly in summer.

### 3.4 GPP estimation based on inversion models

**Figure 9:**
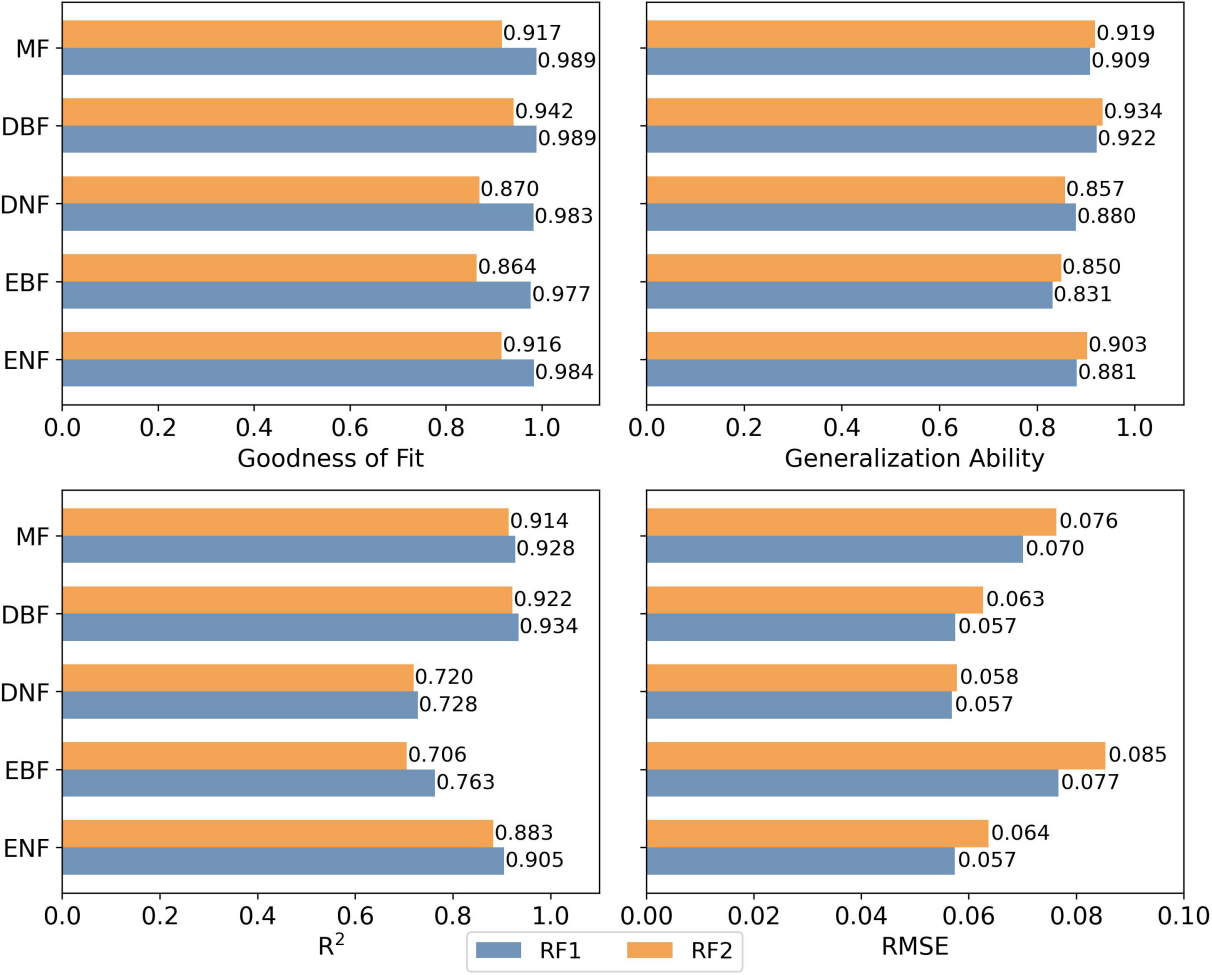
Accuracy results of GPP inversion using RF1 and RF2 models (The difference between RF1 and RF2 models lies in the inclusion of a canopy structural factors in RF1).

To assess the impact of canopy structural parameters on GPP inversion, we compared the accuracy of GPP inversions using different factors, as shown in **Figure 9**. The random forest model incorporating canopy structural parameters (RF1) showed superior goodness of fit (R² ranging from 0.977 to 0.989) compared to the model without these parameters (RF2, R² ranging from 0.864 to 0.942). However, except for DNF, including canopy structural parameters slightly reduced the model’s generalization ability by 1.08% to 2.24%. Incorporating these parameters improved R² by 1.30% to 8.07% and reduced RMSE by 1.72% to 9.41%. Among forest types, DBF had the highest R² at 0.934, and DNF had the lowest at 0.728. DNF also had the lowest RMSE at 0.057, while EBF had the highest at 0.077. Overall, RF1 provided higher inversion accuracy than RF2, indicating that incorporating canopy structural parameters enhances inversion accuracy, though the improvement varied across forest types.

**Figure 10:**
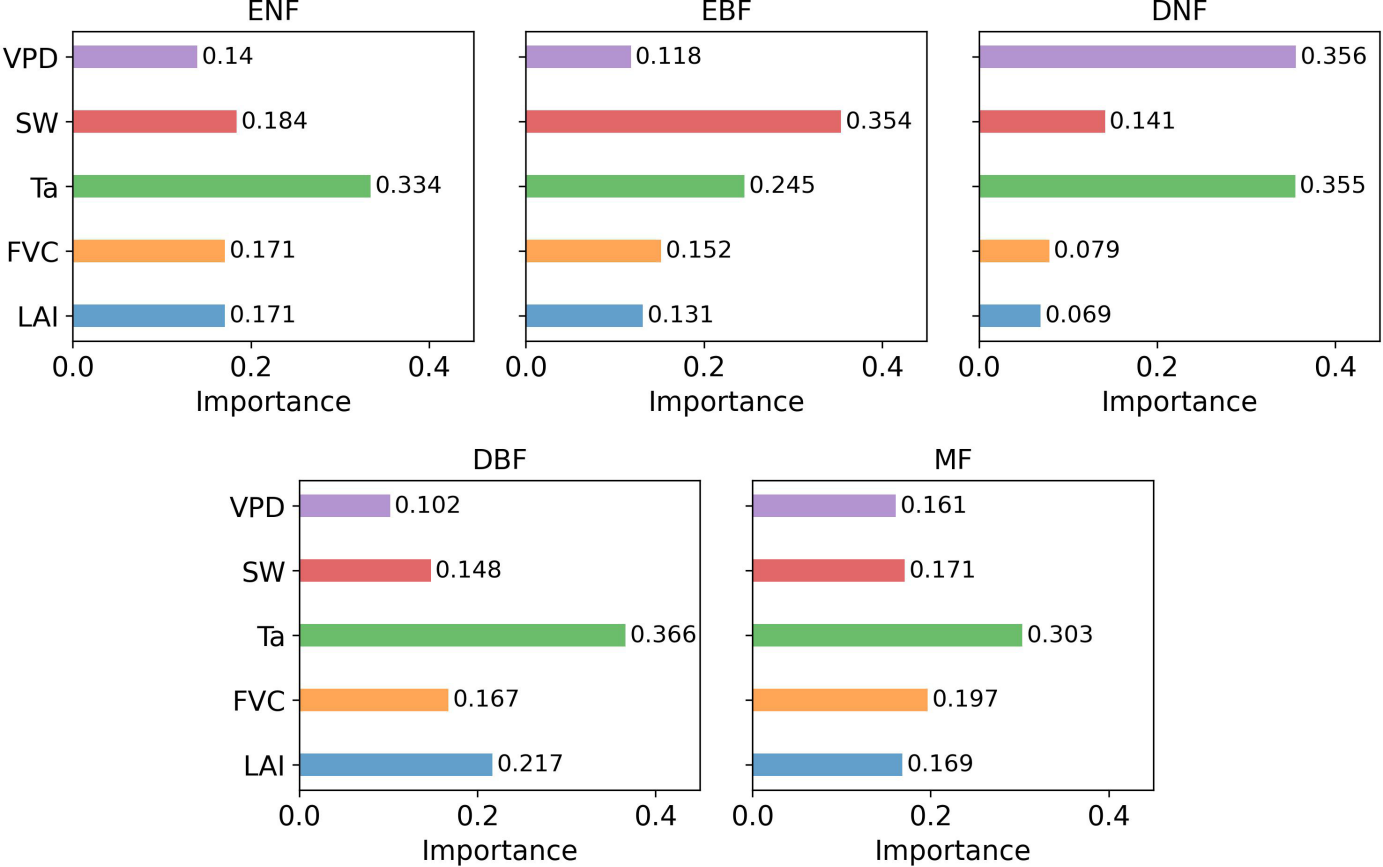
The feature importance results based on the GPP inversion model RF1 for the five forest types.

**Figure 10** illustrated the feature importance for GPP inversion across five forest types, revealing significant variations. Ta consistently showed the highest importance in most models, while VPD was generally the least important. The overall ranking of feature importance was Ta, SW, FVC, LAI, and VPD. In particular, canopy structural parameters (FVC and LAI) had similar contributions, ranging from 0.148 to 0.366, indicating a moderate impact on GPP inversion.

## 4 Discussion

### 4.1 Annual variation of LiDAR canopy structural parameters and data quality

The experimental results indicated that, in the continental United States located in the Northern Hemisphere, canopy structural parameters FVC and LAI, were significantly higher in summer compared to other seasons. The forest canopy exhibited distinct growth and senescence trends in May and October, manifested in corresponding increases and decreases in structural parameter values. Aligned with the Northern Hemisphere’s vegetation growth cycle, these findings coincided with the vegetation’s growth period, a pattern corroborated by previous studies (Sainte-Marie et al., 2014; Tian et al., 2004). The preliminary evidence of the consistency in the forest growth cycle substantiated the efficacy of GEDI data.

**Figure 3** showed that evergreen forests maintain more stable structural parameters year-round than deciduous forests, with less seasonal variation. This aligned with Bonan et al. (2002), which found that evergreen forests exhibit minor greening during the growing season and peak LAI occurs later. Foliation and senescence in forests were mainly driven by photoperiod changes (Sainte-Marie et al., 2014). Evergreen forests exhibited a smaller impact from light response compared to deciduous forests, experiencing a gradual and steady leaf fall throughout the year (Chhabra and Panigrahy, 2011). Consequently, the canopy structure of evergreen forests remained more stable throughout the year. Additionally, broadleaf forests had higher average LAI and FVC than needleleaf forests, with greater seasonal variation, consistent with Liu et al. (2017). These results were mainly attributed to the shorter growing season and the lower solar radiation at high latitudes of needleleaf forest than broadleaf forest. The consistency of LAI and FVC variations across forest types supported the accuracy of GEDI data.

A comparison of MODIS and GEDI canopy structure products (**Figure 4**) revealed a strong and stable correlation (R^2^ = 0.64-0.95) and consistent seasonal variation, with higher values in summer than in other seasons. CDF analysis indicated that GEDI values were generally higher than MODIS, often shifting toward greater values. This difference was more pronounced in certain forest types (e.g., DNF and DBF), where MODIS values appeared more compressed, while GEDI exhibited a more gradual distribution. These discrepancies reflected fundamental differences in measurement techniques, with GEDI capturing more detailed vertical vegetation structure, whereas MODIS provided broader spatial coverage with lower vertical resolution.

### 4.2 Analysis of annual GPP/SIF variation in different forests

The results indicated that GPP/SIF exhibited seasonal variation, being higher in summer compared to other seasons (**Figures 5**, **6**). There has been debate in previous studies about whether the relationship between GPP and SIF shows a consistent linear pattern across different biomes (Li et al., 2018; Liu et al., 2021b; Sun et al., 2017; Wu et al., 2022b). These fluctuations in GPP/SIF confirmed the validity of previous studies (Bai et al., 2022; Chen et al., 2021b), emphasizing the non-constancy of GPP/SIF values across different seasons. The impact of GPP/SIF variability in large-scale GPP estimations cannot be overlooked. The primary factors contributing to these changes include radiation, precipitation, temperature, and structure (Chen et al., 2021a).

Based on the description of GPP and SIF (Damm et al., 2015; Monteith, 1972), GPP/SIF can be described as Equation 4:


(4) $$\begin{array}{c} \frac{GPP}{SIF}=\frac{LUE_{P}}{\Phi_{F}\times f_{esc}} \end{array}$$

where $LUE_{P}$ is light use efficiency of the canopy, ${\text{Φ}}_{F}$ is the chlorophyll fluorescence yield from absorbed sunlight, and $f_{esc}$ is the escape probability of fluorescence. Chen et al. (2021b) suggested that seasonal temperature variations primarily drive seasonal changes in GPP/SIF in temperate regions. Comparing the results with monthly temperature fluctuations in the CONUS in 2020, the most pronounced GPP/SIF changes in June and September coincided with peak temperature variations. The overall annual trend of both curves showed a high degree of consistency, proving the reliability of the study. $LUE_{P}$ and $\Phi_{F}$ were highly correlated to air temperature (Yan et al., 2017) but exhibited different light sensitivities. $LUE_{P}$ showed a saturation pattern under high temperatures, especially on clear days. Notably, differences in SIF and GPP responses to light contributed to discrepancies in spring onset estimates (Yang et al., 2022). In spring, SIF responded to light earlier than GPP, resulting in lower GPP/SIF values. The seasonal non-linear $LUE_{P}$ - $\Phi_{F}$ relationship resulted in a higher $LUE_{P}/\Phi_{F}$ in summer (Kim et al., 2021), contributing to increased GPP/SIF (Bai et al., 2022). In addition, Dechant et al. (2020) reported that the SIF-GPP relationship in crops was dominated by the strongly seasonal $f_{esc}$ rather than the more temporally stable ${\text{Φ}}_{F}$ . $f_{esc}$ has been demonstrated to strongly respond to canopy structural parameters such as LAI (Fournier et al., 2012). In summer, increased LAI and FVC were correlated with a decrease in $f_{esc}$ to some extent, potentially leading to higher GPP/SIF (Zhang et al., 2022, 2020). Comparing two GPP datasets (**Figures 6**, **7**), GPP/SIF trends showed no significant differences, though mean GPP_AMF_/SIF were higher than GPP_MOD_/SIF. This discrepancy likely arose from MODIS GPP underestimation relative to flux tower GPP (Qiu et al., 2020). The standard deviation and annual variation of GPP_AMF_/SIF data were larger, possibly due to the relatively small amount of flux tower data and greater sensitivity to outliers. These findings confirmed the seasonal peak variations in GPP/SIF, reinforcing the study’s conclusions.

### 4.3 Annual correlation between canopy structure and GPP/SIF

The results demonstrated the response of canopy structural parameters, FVC and LAI, to the seasonal variation in GPP/SIF (**Figures 7**, **8**). Overall, FVC-GPP/SIF and LAI-GPP/SIF were moderately well correlated, with this linearity lower in summer compared to other seasons. It confirmed the response of canopy structural parameters to the GPP-SIF relationship.

The relationship between canopy structure and GPP/SIF was complex and lacked a clear mechanistic explanation (Dechant et al., 2020). Absorbed photosynthetically active radiation (APAR) is considered the product of the fraction of absorbed PAR (fPAR) and photosynthetically active radiation (PAR) (Zhang et al., 2018). The relationship between fPAR and LAI can be approximated as (Casanova et al., 1998) Equation 5:


(5) $$\begin{array}{c} fPAR=1-e^{-k\cdot LAI} \end{array}$$

where k is the extinction coefficient. As LAI increased, APAR also increased but gradually saturated at higher LAI due to diminishing increments. High LAI and FVC also reduced the $f_{esc}$ , as multiple scattering and reabsorption within dense canopies limit the amount of fluorescence escaping (Bailey and Fu, 2022; Dechant et al., 2020; Liu et al., 2020; Zeng et al., 2019). High LAI and FVC enhanced APAR (thus increasing GPP) but simultaneously reduced fluorescence escape, suppressing SIF growth, which in turn led to an increased GPP/SIF ratio. Consequently, LAI and FVC were positively correlated with GPP/SIF, as confirmed by **Figures 7** and **8**. Overall, both FVC-GPP/SIF and LAI-GPP/SIF exhibited moderate correlations. However, this relationship weakened in summer, likely due to two main factors. First, higher temperatures can induce stomatal closure and increase transpiration, leading to a decline in light use efficiency ( $LUE_{P}$ ) (Pei et al., 2022), which in turn complicates the relationship between GPP/SIF and canopy structure. Second, in regions with high canopy structural complexity, dense foliage limits radiation penetration to lower leaves, reducing photosynthetic efficiency. As a result, the effect of increasing LAI on fPAR diminished, ultimately constraining GPP/SIF.

Comparing different types of forests, it was observed that deciduous and mixed forests exhibited lower correlations with GPP/SIF in summer than in other seasons. This effect was more pronounced in GPP_MOD_/SIF compared to GPP_AMF_/SIF, possibly due to the larger MODIS dataset reducing the influence of outliers. In summer, the increased canopy structural complexity of deciduous and mixed forests (Hardiman et al., 2011) may weaken the correlation, as higher photosynthetic demand (Bauerle et al., 2012) amplified radiation’s impact on GPP/SIF, reducing the structural influence. In contrast, evergreen forests, with their longer photosynthetic season and lower amortized leaf construction costs (Givnish, 2002), exhibited greater adaptability to light. Their stable year-round canopy structure led to a more consistent impact on GPP/SIF.

### 4.4 The contribution of canopy structural parameters to GPP estimation

GPP estimation results based on RF model indicated that the introduction of canopy structural parameters improved the model’s goodness of fit, but concurrently, it led to a moderate decrease in model generalization ability (**Figure 9**). This suggested that the inclusion of canopy structural parameters enhanced the model’s performance on the training data but reduced its performance on new data. It may be attributed to the high correlation between canopy structural parameter LAI and FVC, as well as the inter-parameter correlations contributing to overfitting, resulting in a slight reduction in generalization ability (Shi et al., 2018). Among the five types of forests, evergreen forests demonstrated slightly higher estimation accuracy compared to deciduous forests. Kim et al. (2021) indicated that ENF exhibits strong seasonal variation in physiology, in contrast to the stable canopy structure. This further underscored the relatively minor influence of canopy structure on ENF or even evergreen forests at a seasonal scale. Furthermore, comparing precision changes after incorporating canopy structural parameters, we found greater accuracy improvement in deciduous forests than in evergreen forests, highlighting a stronger impact of canopy structure on deciduous forests.

The feature importance results indicated that although canopy structural parameters, such as LAI and FVC, responded to changes in GPP/SIF and contributed to improving GPP estimation accuracy, their impact were less significant than that of environmental factors on the GPP-SIF relationship (**Figure 10**). This consistency was observed across diverse forest types. This could be attributed to the more pronounced and impactful spatiotemporal variations in climate at larger scales. Additionally, there was no significant difference in the impact of LAI and FVC on the canopy, which was associated with their high correlation and consistent spatiotemporal variations (Li et al., 2022). The results indicated that canopy structural parameters contributed to the estimation of GPP, but their impact was lower than environmental parameters.

### 4.5 Limitations

In this study, we assessed the contribution of canopy structural parameters obtained from LiDAR to the estimation of GPP. Our research affirmed the spatiotemporal accuracy of LAI and FVC data acquired by LiDAR and their contribution to GPP estimation. However, the limitations of the study included the lack of specific validation of the relationships between parameters such as $LUE_{P}$ , $f_{esc}$ , and ${\text{Φ}}_{F}$ that influence the GPP-SIF relationship, and canopy structural parameters. Additionally, current research on the relationship between GPP/SIF and canopy structural parameters was limited to correlational analyses, without establishing the theoretical causal relationships between the parameters. Furthermore, for large continental spatial scale, many elements contributed to the uncertainty of GPP estimation (Tramontana et al., 2015). Reducing GPP uncertainty at large scales and improving estimation accuracy remained an area for further investigation. The limitations of this study suggested avenues for future improvement.

## 5 Conclusion

In this study, we integrated spaceborne LiDAR (GEDI) and other multi-source datasets to examine the response of canopy structural parameters to the GPP-SIF relationship and their impact on large-scale GPP estimation in forested ecosystems. Specifically, we: evaluated the capability of GEDI-derived canopy structure products to capture vegetation dynamics; investigated the response of canopy structural parameters, LAI and FVC, to the GPP-SIF relationship; and quantified the contribution of canopy structure to GPP estimation using machine learning approaches. The main findings are as follows:

1. LiDAR-derived canopy structural products effectively captured seasonal dynamics and structural variations across different forest types. Compared to MODIS, they exhibited strong correlations and similar temporal patterns. However, in densely vegetated areas, LiDAR yielded higher LAI and FVC values, highlighting its enhanced capability to resolve vertical vegetation structure.
2. GPP/SIF showed significant seasonal variations in all forest types except EBF, with higher values in summer.
3. Canopy structural parameters, LAI and FVC, showed seasonal variation in their correlation with GPP/SIF (correlation coefficient: 0.21-0.75). In summer, the correlation with GPP/SIF was lower in deciduous and mixed forests, decreasing by 5.53% to 30.59% compared to other seasons.
4. The inclusion of canopy structural parameters improved the accuracy of model-based large-scale GPP estimation, increasing R² by 1.30% to 8.07%.

## Data Availability

The original contributions presented in the study are included in the article/supplementary material. Further inquiries can be directed to the corresponding author/s.
